# Fatigue Resistance of Bituminous Mixtures and Mortars Containing High Reclaimed Asphalt Content

**DOI:** 10.3390/ma13245680

**Published:** 2020-12-12

**Authors:** Alexandros Margaritis, Geert Jacobs, Georgios Pipintakos, Johan Blom, Wim Van den bergh

**Affiliations:** 1 EMIB Research Group, University of Antwerp, 2020 Antwerp, Belgium; alexandros.margaritis@uantwerpen.be (A.M.); geert.jacobs@uantwerpen.be (G.J.); georgios.pipintakos@uantwerpen.be (G.P.); johan.blom@uantwerpen.be (J.B.); 2 Belgian Road Research Center, 1200 Brussels, Belgium

**Keywords:** reclaimed asphalt, bituminous mortar, asphalt, fatigue resistance, dynamic shear rheometer, four-point bending, dissipated energy

## Abstract

With the increased use of reclaimed asphalt (RA), the ability of bituminous materials to resist fatigue cracking may face a decline mainly due to the aged reclaimed asphalt binder (RAB), especially when RA is used at higher rates and not sufficiently treated. In this study, the bulk scale (asphalt) and its subscale (mortar) were employed to evaluate the effect on fatigue resistance when a RAB is added, by considering three replacement rates: 0%, 40%, and 70% RAB. The fatigue testing of asphalt mixtures was carried out using a four-point bending (4PB) setup, while the mortars were tested using a new column-like geometry utilising a dynamic shear rheometer (DSR). The fatigue properties were further analysed using dissipated energy concepts. The aim of this study was, first, to assess whether the inclusion of a RAB can provide at least similar fatigue properties compared to an all-virgin mix, and second, to evaluate whether the proposed treatment is beneficial for the mixtures with a RAB. The asphalt tests revealed that the inclusion of a 40% RAB led to increased fatigue resistance, whereas the mortar tests showed that the inclusion of RAB has an inverse effect on fatigue life.

## 1. Introduction

At present, asphalt recycling is prevalent within the road construction industry because it is accepted as a sustainable approach. Although the economic and environmental benefits of using reclaimed asphalt (RA) are widely accepted, questions arise from the construction sector concerning the service life of asphalt mixtures with RA. In Belgium, the current average content of RA in new mixtures is 40%, and this is almost exclusively used in dense-graded asphalt mixtures for base layer applications [1]. According to recent studies, this RA content appears to perform reasonably well in the lab and has a good environmental impact [1,2], but there is certainly the potential to go higher in terms of recycling rates.

Concerning the effect of RA on the performance of asphalt mixtures, one of the main engineering problems associated with the inclusion of RA in base layer asphalt mixtures is fatigue cracking [3,4,5,6]. One of the most critical distress types for asphalt pavements is cracking. The focus of this study will be on the cracking done at intermediate temperatures or also known as fatigue cracking. This type of cracking initiates when excessive tensile strains develop at the bottom of the asphalt layer leading to crack initiation [7].

In the US, many studies have been performed concerning the field performance of mixtures containing RA. In 1991, the FHWA initiated the Long-Term Pavement Performance (LTPP) programme, which provided valuable results based on field performance observations [8,9]. Overall, it was concluded that the addition of up to 30% RA did not alter the performance of mixtures significantly. In a project conducted by the California Department of Transportation, it was concluded that after five years of service, no difference was observed between the virgin and recycled mixtures (15% RA) [10]. Generally, the abovementioned field results can be considered reasonable, taking into account the rather conservative recycling rate, with the highest being 30%.

Several studies have attempted to evaluate the effect of high RA, i.e., above 40%, in the lab. Some lab-based studies showed a positive effect of RA, with some indicative examples being the studies of Al Qadi (up to 50% RA) and McDaniel (up to 40% RA) [5,11]. Margaritis et al. statistically analysed fatigue results of 74 lab-tested mixtures, used in Flanders. The results showed that mixtures with RA (up to 60% RA) exhibited similar fatigue properties compared to virgin mixtures [1]. Furthermore, Sabouri et al. concluded that the inclusion of 40% RA led to reduced resistance in fatigue cracking [12]. A study by Mangiafico et al. showed that fatigue resistance was higher for mixtures with RA from 20% until 40% and that for mixtures with 60%, a small drop was observed, concluding that the optimum percentage in terms of fatigue resistance is between 20% and 40% [13].

Typically when fatigue is evaluated in the lab, the focus is on the bulk scale, i.e., the asphalt mixture. Over the years, many studies have experimentally evaluated certain mechanical responses, such as fatigue, not only in the bulk scale but also in the subscales, such as the mortar scale, which represents the mesoscale. This phase is crucial for the bituminous mixture, as two failure types will initiate from it, namely cohesive and adhesive failure. Testing bituminous materials in a subscale is not uncommon. Fatigue tests in the binder scale are very typical for benchmarking and classifying different bituminous binders. Moreover, testing bituminous mastics (bitumen and filler) has also been widely used, and this has the advantage of including the filler, where different filler types can greatly influence their fatigue behaviour [14,15]. The mortar scale also allows for the inclusion of sand (the whole fraction or part of it), which has been demonstrated to play a significant role in the bituminous coating and the bonding of the coarse aggregate skeleton [16,17].

## 2. Objectives and Scope

To assess the true effect of RA, it is necessary to evaluate mixtures with very similar mix design and binder properties. The primary objectives of this paper were two-fold. The first is to assess whether the inclusion of RA can provide at least similar fatigue properties compared to an all-virgin mortar and asphalt mix. The second is to evaluate whether adding only the virgin binder, without the use of other recycling agents, is beneficial for the mixtures with RA. In this study, three mixtures with 0%, 40%, and 70% reclaimed asphalt binder (RAB) are designed and evaluated. Asphalt beams are tested using a four-point bending (4PB) setup and column-like mortar samples using a dynamic shear rheometer (DSR). The results are analysed using conventional criteria and fundamental energy concepts.

## 3. Materials

To evaluate the effect of RAB on the fatigue resistance, three asphalt concrete mixtures with a nominal maximum aggregate size of 14 mm (AC14 or APO-B according to the Flemish road regulations) were theoretically designed by considering three replacement rates: 0%, 40%, and 70% RAB. The RA was provided by a local asphalt plant, and it is a homogeneous (milled from a single road source), continuous graded 0/14 mm asphalt mixture with a 5.18% binder content (on mixture mass). The RAB has a penetration of 24 0.1 mm and a softening point of 63 °C, measured based on EN 1426 and EN 1427 [18,19], accordingly. More information on the chemical and rheological properties of the RAB can be found in a previous study [20], where the RAB is denoted as the RA18 sample.

The gradation of the three mortars was derived from the corresponding asphalt mixtures. The mix design of the bituminous mortars and mixtures was similar in terms of aggregate gradation and binder blend penetration, contingent upon the assumption of full blending, a representative of the standard practice which, however, may not be valid as demonstrated by previous studies.

**Figure 1:**
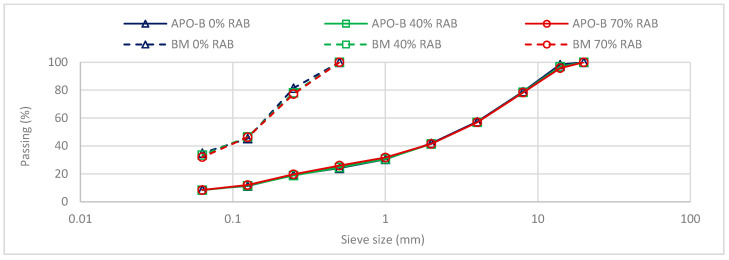
Particle size distribution of the bituminous mortar (BM) and asphalt mixture (APO-B) designs for the three reclaimed asphalt binder (RAB) replacement rates: 0%, 40%, and 70% RAB.

All asphalt mixtures were designed theoretically using the design software PradoWin (Belgian Road Research Centre, Brussels, Belgium), based on a typical design of an AC14 mixture used in Flanders. The mixtures were designed with a theoretical binder content of 4.3% on aggregate mass (4.12% on mixture). The gradation curves of the mixtures and mortars are presented in Figure 1. The fractional composition of the bituminous mortars and mixtures is given in Table 1. Furthermore, the maximum density was calculated according to EN 12697-5 [21], and then, for the tested asphalt beams, the air voids were estimated while considering the volumetric estimation of the bulk density. The maximum density and the average air voids of the three asphalt mixes are presented in Table 2.

The production of asphalt mixtures was carried out following the EN 12697-35:2016 [22]. For the 40 and 70% RAB mortar mixtures, the RAB was extracted and recovered and consequently mixed with virgin binders, providing the necessary binder blends for the mortar mixtures. It must be noted here that the used mortar composition of this study followed an enriched composition, which is necessary to achieve a workable and thus self-compactable mortar mix. An enriched composition means that the mortar mixtures have more bitumen than the actual mortar phase in the bulk. The procedure to calculate the enriched composition, as well as the fabrication steps of mortar mixes, are elaborately discussed and presented in a previous study [23]. Finally, it must be noted here that the proposed fabrication method aims to minimise the air voids in the mortar sample. The reason for that is to achieve a homogeneous material and to assess its mechanical performance (i.e., fatigue) without the existence of air cavities within the sample, which may influence the integrity of the sample and thus its fatigue resistance.

To achieve the same binder properties between the mixtures, the binder blend cases were theoretically designed, using the log-pen rule in Equation (1), provided by EN 13108-1 [24]. For the control mixture with 0% RAB, a 35/50 penetration grade bitumen was used, with a penetration value of 43 0.1 mm. The penetration of the control virgin binder was used as the base for the second and third binder blends, i.e., 40% RAB and 70% RAB, respectively, and it was developed using Equation (1). To achieve the exact same penetration, two new artificial binder blends were developed for the 40% and 70% RAB mix, namely a hard blend (HB) and soft blend (SB), respectively. The exact compositions are presented in Table 3 and Table 4. Furthermore, the measured penetration and the softening point of the final binder blends are presented in Table 3.

(1) $$a\log\left({\mathrm{pen}}_{\mathrm{virgin}}\right)+b\log\left({\mathrm{pen}}_{\mathrm{RAB}}\right)=\log\left({\mathrm{pen}}_{\mathrm{mix}}\right)$$

Factors a and b are the proportions of the two components (a + b = 1); pen_virgin_, pen_RAB_, and pen_mix_ are the penetration values of the virgin binder component, the RAB component, and the final binder blend mix, respectively.

## 4. Experimental Investigation

### 4.1. Fatigue Testing

**Figure 2:**
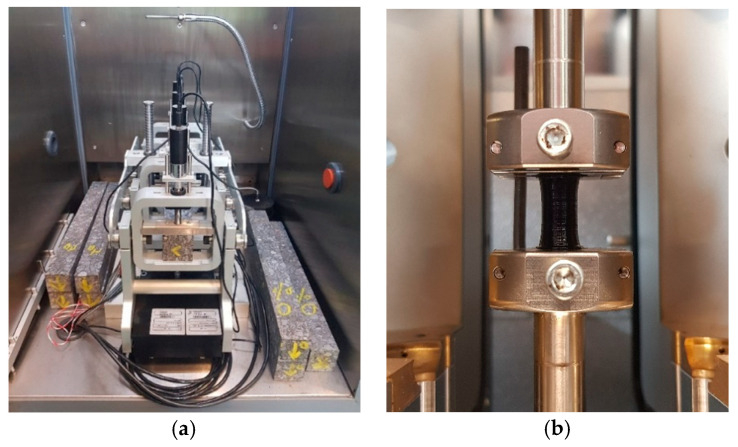
Four-point bending (4PB) testing setup for the asphalt mixtures (**a**) and clamping of the bituminous mortar inside the dynamic shear rheometer (DSR) temperature chamber (**b**).

The asphalt fatigue tests were performed using the 4-point bending (4PB) setup, and the mortar tests were done using a DSR (see Figure 2). Two key parameters were defined for all three mixtures, i.e., their stiffness modulus and fatigue resistance. Both tests were performed using the 4PB testing setup, as described in EN 12697-26:2018 (Annex B) and EN 12697-24:2018 (Annex D), respectively [25,26]. To define the |E*| modulus, cyclic tests were performed at 15 °C, and their total duration was set for approximately 1 min, per frequency step (0.1 to 20 Hz). The fatigue tests were performed under a strain-control mode, between 85 and 145 μm/m, at 15 °C, and 30 Hz. For the fatigue characterisation of each mixture, at least 3 replicates were tested at a minimum of 3 strain levels, with the total number of replicates ranging from 11 to 15.

For the evaluation of the fatigue resistance of bituminous mortars, a newly proposed sample geometry was utilised [23]. The mortar sample geometry consists of three parts, namely two half one-sheeted hyperboloids, both approaching the diameter of a cylindrical middle part. The mortar samples were tested using torsional time-sweep tests in an oscillatory mode, and the measurements were performed until the complete failure of the specimen. The performed torsional tests were stress-controlled and not strain-controlled since stress is typically more accurately and faster achieved, given that most DSR devices are torque-controlled. The stress levels were selected with the purpose of keeping them outside of the linear viscoelastic region (LVER). This was the case for both asphalt and mortar fatigue tests. Therefore, the mortar mixtures were tested using stress amplitudes between 0.85 and 1.40 MPa, and the tests were performed at 15 °C and 10 Hz. For each mortar mix, 17 to 24 samples were tested. More information concerning the fatigue testing framework, the used geometry, and fabrication procedure are presented in a previous study [23].

### 4.2. Fatigue Resistance Analysis: Conventional and Energy Concepts

The fatigue resistance of the tested asphalt mixtures was further analysed based on two approaches: using the empirical failure criterion N_f50_ and using energy approaches. The energy concepts are based on the fundamental response of the material when sustaining external loading. During cyclic loading, two curves are generated on a stress–strain plot, representing the loading and unloading phase of the material. This plot is also known as the hysteresis loop, and its area provides the dissipated energy (DE) per cycle, which can be approximated using Equation (2).

Many energy concepts that make use of the DE approach have been introduced. The method that is possibly the least unbiased one is the ratio of dissipated energy change (RDEC). The RDEC approach was introduced to provide a better approximation of the actual damage, taking into account only the DE change between the cycles [27,28]. RDEC can be calculated using Equation (3). When the RDEC is plotted against the loading cycles, a plateau energy stage can be observed. This plateau stage expresses a unique energy parameter, namely the plateau value (PV). It has been demonstrated that the PV exhibits an excellent relationship with the N_f50_, which is independent of temperature effect, material properties, and loading mode [28,29].

(2) $$w_{i}={\pi \sigma }_{i}\varepsilon_{i}\sin(\delta_{i})$$

(3) $${\mathrm{RDEC}}_{b}=\frac{w_{b}-w_{a}}{w_{a}\left(b-a\right)}$$

where ε is the strain (m/m); σ is the normal stress (kPa); δ is the phase angle (^o^); $w_{i}$ is the dissipated energy per cycle I (kj/m^3^); a and b are cycles with b > a; ${\mathrm{RDEC}}_{b}$. is the ratio of dissipated energy change at cycle b during a cycle period of b − a cycles; and $w_{a}$ and $w_{b}$. are the dissipated energies at cycles a and b, respectively.

For the mortar fatigue assessment, the phenomenological approach N_f,G*xC_ was used first to assess the fatigue resistance of the mortar. The phenomenological failure was estimated by plotting the product of G* and cycles against cycles, and then a distinctive maximum point was observed (N_f,G*×C_) [30]. In addition to the N_f,G*xC_ approach, two energy concepts were also considered: the dissipated energy ratio (DER) and the RDEC concept. For the DER approach, fatigue was derived between the N_p20_ (i.e., the moment where the DER deviates more than 20% from the linear viscoelastic state) and the W_0_, the initial DE. For the mortar samples, the DE per cycle was calculated using Equation (2), taking into account the shear stress (τ) instead of the normal stress (σ). In a previous study, it was demonstrated that the energy parameter PV correlated well with all failure moments N_f50_, N_f,G*xC_, and N_p20_, which provided a unique fatigue model, with the best fit being achieved for the model between PV and N_p20_ [23].

One of the discouraging points of the RDEC approach is the rather challenging estimation of the PV value. Carpenter and Shen defined an equation, based on which the PV can be estimated [28]. First, a power fit must be established between the DE and loading cycles (LC): DE = αLC^f^, with a and f being the regression coefficients of the fit. Based on the slope f and the N_f50_ of the tested sample, the PV can be estimated. However, fitting a power fit is not always successful, as previously stated also by Subhy et al. [31], and therefore calculating the PV with the formula Shen and Carpenter proposed is not always feasible. To counteract for this issue, the following methodology is proposed to extract a more reliable PV. First, by visual inspection, PV is defined by checking in which RDEC phase (II or III) the N_f50_ falls in. Then, if the N_f50_ falls within phase II, the PV is calculated as the average RDEC of the 10 last points before N_f50_. If N_f50_ falls in phase III, then the PV is calculated by taking the 10 average RDEC points before the midlife point of N_f50_ (0.5 × N_f50_), which for all tested samples fell in phase II.

**Figure 3:**
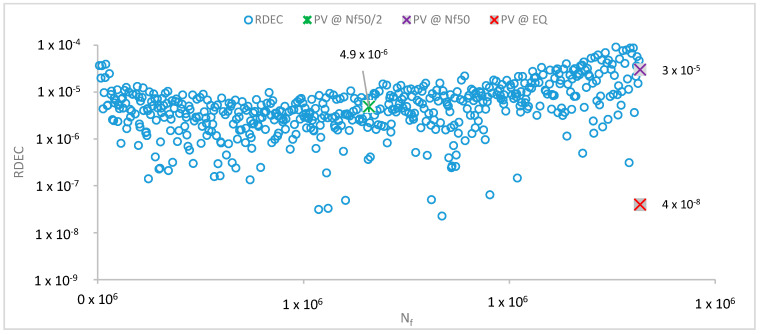
Estimation of the plateau value (PV) using the ratio of dissipated energy change (RDEC) plot against the loading cycles.

As an example, three PV values were calculated and presented in Figure 3. First, the PV was estimated using the Carpenter and Shen equation (PV@ΕQ). Then, the PV was calculated from the average of 10 RDEC points before N_f50_ (PV@N_f50_), and finally, the average of 10 RDEC points before 0.5 × N_f50_ was used for PV (PV@N_f50/2_). For this sample, the N_f50_ fell within phase III. Consequently, the PV@N_f50_ seems to be overestimated (3.0 × 10^−6^), since phase III has already started, and the RDEC values are increased. In contrast, the PV@ΕQ is underestimated (4.0 × 10^−8^), being at the same level as some (very few) RDEC points, which seem to be more like outliers. Finally, the PV@N_f50/2_ (4.9 × 10^−6^) is right within the cloud of data points of the plateau stage, indicating that it is a more appropriate estimation.

## 5. Results and Discussion

### 5.1. Bituminous Mixtures

**Figure 4:**
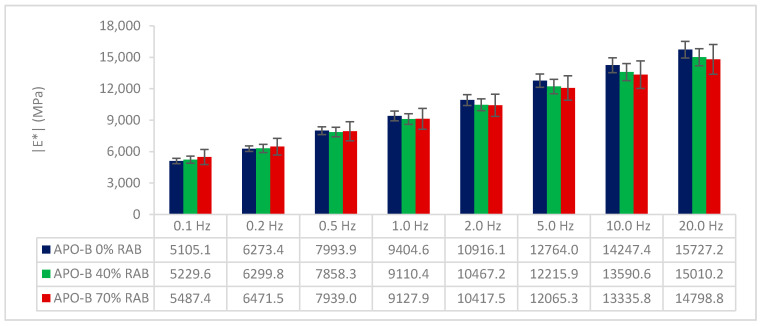
|E*| modulus of the three mixtures under various frequencies.

The |E*| modulus of the 3 mixtures at 15 °C and at a frequency range from 0.1 to 20 Hz, is illustrated in Figure 4. Starting at the lowest tested frequency (0.1 Hz), the mixtures with RAB showed a slightly higher |E*|, and at 0.2 Hz the |E*| was similar among the 3 mixtures. Above that frequency point, the mixtures with RAB showed a gradually small decrease in |E*|, as frequency increased. It can be observed that the 70% RAB mix shows the highest deviation compared with the rest, and this can be attributed to the high replacement rate, which induces a certain variability. Nevertheless, the observed small differences cannot be characterised as significant since there are overlapping standard deviations, and thus it can be concluded that the three mixtures demonstrate a similar |E*| modulus. These results are rather expected since the gradation and the binder penetration are similar, at least theoretically, as well as the average air voids of the asphalt beams. Therefore, the addition of RA seems to not have an influence on the |E*| modulus of the studied mixtures.

**Figure 5:**
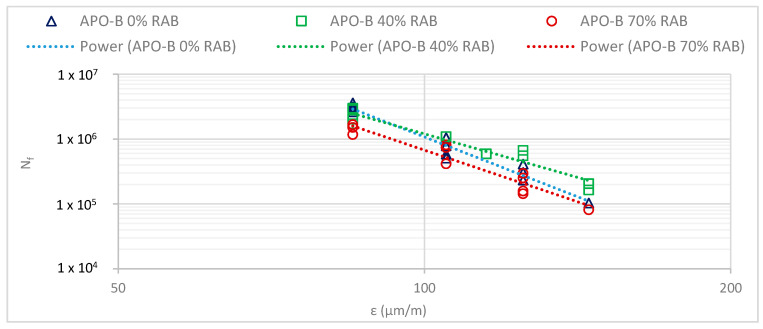
Fatigue curves (Nf50) of the three asphalt mixtures.

To assess the effect of RAB inclusion on the fatigue resistance of dense-graded asphalt mixtures, both conventional and energy criteria were considered. The fatigue life can be depicted using the Wöhler curves, where the applied strain is plotted against the cycles until failure (N_f_). The fatigue curves are presented in Figure 5, and the parameters of the fatigue curves are described in Table 5.

The ε_6_ parameter represents the strain level at which the fatigue life is 10^6^ cycles and is widely used as a fatigue resistance parameter. A higher ε_6_ indicates a higher fatigue life. For this study, the 40% RAB mix exhibited the highest fatigue resistance, followed by the 0% RAB mix. The mixture with the 70% RAB showed the lowest fatigue resistance. These findings are in line with previous studies that have stated that with up to a 40% RA inclusion, the fatigue resistance is expected to be higher and then a turning point occurs, above which the trend is different [13].

However, ε_6_ is only a single point, which may not provide a universal fatigue ranking among the mixtures, since in reality, different strain levels may appear at the bottom of the base asphalt layer. Taking a different strain level as an example, i.e., the ε_5_ point, the 40% RAB mix still shows the best performance but with a substantially larger difference compared to the second-best mixture, the 0% RAB. At the ε_6_ point, the difference in strain level between the two mixtures (0% and 40% RAB) was only 2 μm/m, whereas the difference at the ε_5_ point was 35 μm/m. This points out the significance of taking into account the slope of the fatigue curve also.

The three mixtures tested in this study exhibited different fatigue slopes, with the 0% RAB mix having the steepest fatigue life slope. Contrary to this investigation, a number of previous studies have reported that ageing will lead to steeper slopes [32], as well as the inclusion of RA, which will lead to similar results (50% RA) [33]. Other studies have reported similar slopes between all-virgin mixes and mixes containing RA (up to 40% RA) [12,34]. Mangiafico et al. claimed that when the two binder parts, i.e., RAB and virgin, have a similar penetration, the slope of the fatigue mixture will not be influenced significantly [35]. Based on this statement and considering the similar theoretical penetration between the blends in this study, it is possible that the fatigue slopes in Figure 5 were influenced by the presence of softer binder zones or thinner binder coating for some aggregates (possibly the virgin aggregates).

As it is evident, the selection of the virgin bitumen and the RAB content will significantly affect the fatigue properties [1]. The findings of the fatigue investigation point out the possible existence of a “softer” binder blend, which is apparent for the 70% RAB mix. This was not the case for the stiffness measurements, where the |E*| was similar among the mixtures. It is, however, possible that the inclusion of RA led to heterogeneous zones within the mixtures, with different binder blend stiffness and binder coverage. These zones are especially crucial during repeated loading (fatigue) since they will act as weaker zones. More specifically, the stiffer inactive RAB zones are expected to build higher stress concentration zones that are prone to earlier cracking. The higher the replacement rate, the higher the risk for such zones, especially evident at the 70% replacement rate. It is likely that by increasing the recycling rate, the risk of having a lower degree of activation is higher. In addition, the inclusion of a very soft virgin binder does not have a beneficial effect on the RAB activation, as demonstrated by the results of the 70% RAB mix.

In a previous study, it was concluded that the actual bituminous coating of RA is a bituminous mortar, as demonstrated by microscopic images [16]. Commonly for conventional bituminous binders, certain viscosity levels are defined to allow them to adequately mix and cover the aggregates in a bituminous mixture. This viscosity level (0.17 ± 0.02 Pa·s) is achievable at temperatures between 135 and 180 °C depending on the bitumen type, as described in Table 1 of EN 12697-35:2016 [22]. The viscosity of a field-aged binder is expected to be much higher since it is not a pure bituminous binder but rather a bituminous mortar. Because the preheating temperatures are limited, to avoid further ageing of the RAB, the RA material does not have enough heat capacity to enable its binder. Hence, the use of a rejuvenator is recommended, especially at higher RAB replacement rates (above 50%). It has been previously demonstrated that the rejuvenator can act beneficially in the diffusion process between the virgin and aged binder [36,37].

For lean mixtures, meaning low binder content mixtures (like the 70% RAB mix of this study), there is a considerable risk of ending up with virgin aggregates poorly covered by bitumen. This risk is even higher at high recycling rates, considering the possibility that the RAB activation can be rather low and that the biggest part of the existing bitumen already covers the RA aggregates. This might create a possible weak spot in the mixture at the virgin aggregates since there is not enough bitumen coverage. This problem might be avoided by increasing the total binder content, consequently compensating for the possible inactive RAB. Finally, mixtures containing high RA content should be designed following the performance-based mix design principles, since by adopting this approach, the potential implications of partial blending will be revealed by their effect on the mechanical performance [38].

**Figure 6:**
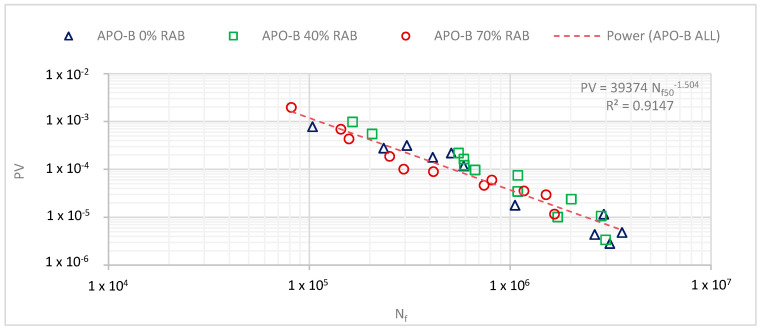
Unified fatigue model for all three mixtures, based on the plateau value (PV) and the loading cycles to failure (N_f_).

Besides the conventional failure criterion N_f50_, the fatigue results were analysed using the RDEC approach. Based on the approach described in Section 4.2, the PV was calculated for all tested samples, and then plotted against the N_f50_. The results showed a unified model between the three mixtures (Figure 6). Based on the unified fatigue curve, it is impossible to distinguish and benchmark the three mixtures. For that reason, a different approach that attempts to relate the structural design with the actual fatigue resistance is proposed.

Typically, during the design process of a pavement structure, the strain levels are estimated at different depths, with a great focus on the bottom side of the asphalt layer, since it is the spot where maximum tensile stress is expected to appear. For this reason, two tested strain levels were selected: 85 and 125 μm/m, in an attempt to represent two structural scenarios: a “thicker layer” and “thinner layer” scenario. This is based on the assumption that under the same loading conditions, the two structural scenarios (i.e., thicker and thinner layers) will experience a lower and a higher strain level condition, respectively. Rearranging the power fit from Figure 6, a new fatigue model was derived for the three mixtures that is able to calculate the N_f_ for a given PV (Equation (4)). The two strain levels were also selected on the basis that at least three replicates were measured for those two strains.

(4) $$N_{f}=1938.7{\mathrm{PV}}^{-0.608}.$$

One should note here that all three mixtures have similar |E*| at 15 °C and 10 Hz (in the range of 13,300–14,200 MPa), a metric typically requested for design calculations. Additionally, more research is needed to define the representative strain levels for standard layer thicknesses. In this study, to benchmark the materials based on their PV, two strain levels were selected based on a lower and a higher strain level, representing a thicker and a thinner structure.

**Figure 7:**
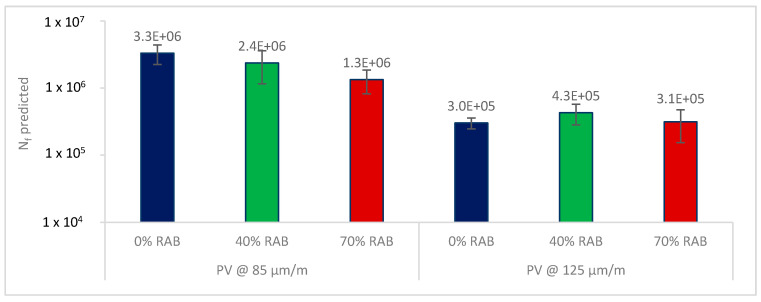
Predicted fatigue life of the asphalt scale, per mixture, for the two strain levels based on the PV (Equation (4)).

Based on the defined fatigue life, it is expected that the lower the PV is, the longer the expected fatigue life is. For every PV point, a new N_f_ was estimated using Equation (4). The average values of the predicted N_f_ are illustrated in Figure 7, per mixture and strain level. Based on the findings, at 85 μm/m, the 0% RAB mix showed the highest fatigue resistance, whereas at the 125 μm/m level, the 40% RAB mix had a better fatigue life. The latter agrees with the conventional fatigue parameter ε_6_. It must be noted here that the standard deviations for the three mixtures are overlapping. As a result, it can be speculated that the average values are not statistically different. However, this cannot be further statistically tested because of the limited number of replicates.

### 5.2. Bituminous Mortars

**Figure 8:**
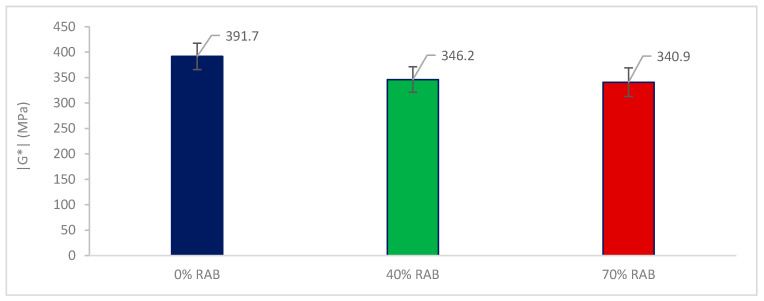
|G*| at 15 °C and 10 Hz for the three mortar mixes.

Figure 8 shows the |G*| for the mortar samples, tested at 15 °C and 10 Hz. Prior to fatigue testing, the mortar samples were tested at a low stress level within the LVER (0.001 MPa) in order to measure the |G*| of the mortar samples. The results show that the 0% RAB mortar mix has the highest |G*|, followed by the 40% and 70% mixes. Unlike the asphalt mixture results where the three mixtures demonstrated similar modulus, the mortar mixtures with 40% and 70% RAB showed a lower modulus on the mortar scale. Finally, the addition of a different filler type in the 40% and 70% RAB mortar mixes, i.e., the one coming from the RA, seems to have no impact on the measured |G*|, since these results follow the results of the measured penetration for the three final binder blends with 0%, 40%, and 70% RAB.

**Figure 9:**
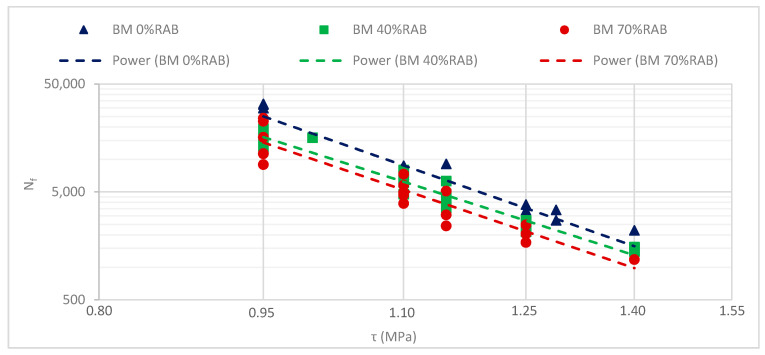
Fatigue curves of the three bituminous mortar mixtures, based on the N_f,G*xC_ criterion.

Next, the fatigue results of the phenomenological approach are depicted in Figure 9, and the regression parameters of the fatigue lines are shown in Table 6. According to the N_f,G*xC_ criterion, the inclusion of RAB leads to a decrease in fatigue life, and hence the all-virgin mortar (0% RAB) exhibits the highest fatigue resistance. The results of the mortar scale seem to be strongly influenced by the stiffness of the samples, as they follow the same ranking as the |G*| of the mortar samples.

The N_f,G*xC_ fatigue results are not in line with the results of the asphalt-scale fatigue tests, according to which the 40% RAB mix exhibited the highest fatigue resistance based on the conventional fatigue criterion ε_6_. However, attempting to relate the two scales should be done with care, considering the specific differences between them.

Firstly, the failure type between the two scales is different. For the asphalt scale, both adhesive and cohesive failure may take place, whereas in the mortar scale, only the resistance against cohesive failure is considered. In fact, due to the presence of the fine particles, this failure mode can also be considered as semicohesive, since the loss of adhesion between the bituminous binder and the fine particles may also occur. However, since it is regarded as one phase that covers and bonds the coarse skeleton, the primary failure type that drives this phase (i.e., the mortar) to fail is considered to be cohesive. Secondly, the deformation mode is different; the mortar samples are deformed in shear mode while asphalt samples are in tension–compression. Finally, the two scales were tested under a different loading mode, namely the asphalt-scale tests under strain control and the mortar under stress.

**Figure 10:**
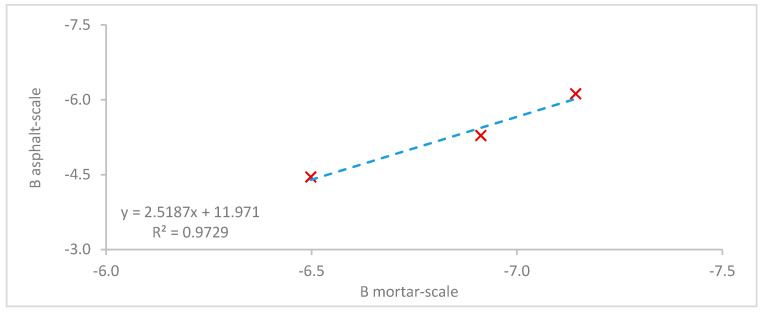
Relationship between the slopes (B) derived from the mortar- and the asphalt-scale fatigue tests.

Nevertheless, a linear relationship can be obtained between the slopes of the fatigue tests, as depicted in Figure 10. To further support this relationship, more tests are necessary as well as the consideration of different materials. Finally, the effect of the enriched binder content should be investigated in more depth to further solidify the relationship to the asphalt scale.

**Figure 11:**
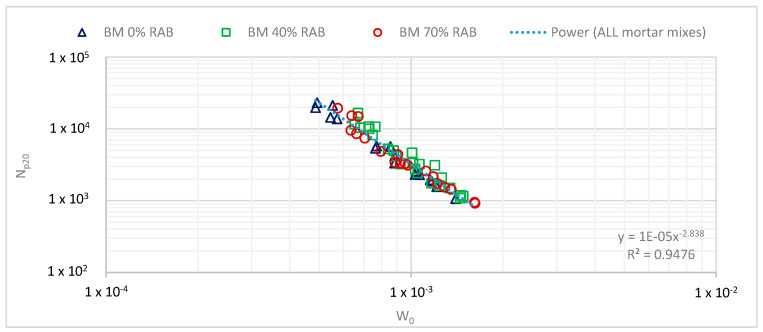
Fatigue resistance of mortars, based on the DER approach (N_p20_).

The fatigue resistance of mortars was further evaluated using the energy concepts of DER and RDEC. In an earlier study concerning the fatigue characterisation of mortars, it was found that the DER approach led to a fatigue model that was independent of temperature but dependent on the bitumen type (modified or not) [23]. Figure 11 presents the result of the DER approach for the studied mortar samples. Here, a unified model was derived, which was expected since none of the mortars contained a polymer-modified binder (PMB).

**Figure 12:**
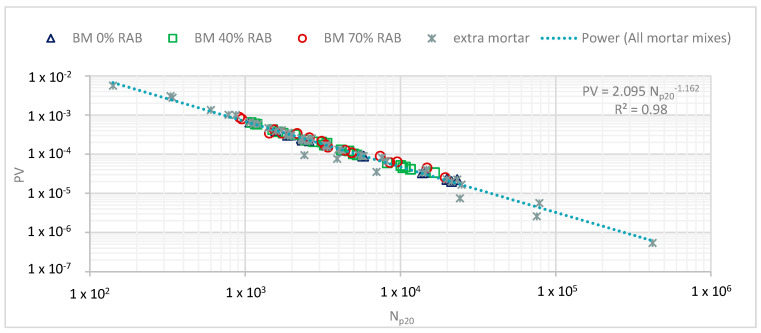
Fatigue resistance of the mortars for the three replacement rates of 0%, 40%, and 70% RAB based on the RDEC approach: Np20 vs. PV. The extra mortar points were derived from a previous study [23].

The obtained N_p20_ for every tested mortar sample was used together with the findings of the RDEC approach. Figure 12 illustrates the unified fatigue model, using the results of the 0%, 40%, and 70% RAB mortar mixtures. Furthermore, extra mortar samples (presented in a previous study [23]) were additionally considered, i.e., mortars containing PMB and mortars tested at different temperatures (different than 15 °C) as well as different loading modes, thus leading to an even more robust fatigue model.

Following the same approach as for the fatigue tests performed in the asphalt scale, two tested stress levels were selected to represent two structural scenarios: the 0.95 and 1.25 MPa, representing a thick layer and thin layer, respectively. The two stress levels were the lowest and highest tested stress levels with at least 3 repetitions. Rearranging the resulted fit of Figure 12, a new fatigue model is derived for the three mortars that is able to estimate the N_f_ for a given PV (Equation (5)).

(5) $$N_{f}=2.1982{\mathrm{PV}}^{-0.844}$$

**Figure 13:**
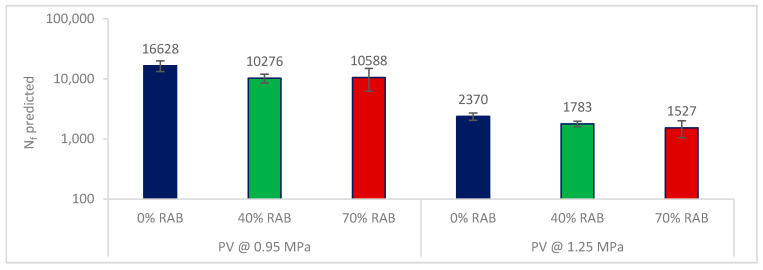
Predicted fatigue life of the mortar scale, per mixture, for the two stress levels based on the PV (Equation (5)).

For every single PV value, the corresponding N_f_ was calculated using Equation (5). The average predicted N_f_ values are illustrated in Figure 13 per mortar and stress level. For both layer structures (thicker and thinner layer), the 0% RAB mix showed the highest fatigue resistance, as this was also demonstrated based on the N_f,G*xC_ approach. Similarly to the asphalt scale results, the predicted N_f_ exhibited overlapping standard deviations, which possibly indicates nonsignificant differences among the average values. This cannot be further statistically verified as it requires a larger number of replicates.

The ranking of the estimated fatigue life was similar to the one derived from the asphalt scale for the thick-layer case (PV at 85 μm/m). For the higher stress level of 1.25 MPa, the mortar fatigue is more severe for the samples with lower |G*|, i.e., the samples with 40% and 70% RAB, showing the lowest fatigue resistance. One of the disadvantages of a stress-control test is that the samples may fail due to excessive creep deformation, and hence a “softer” sample will exhibit lower fatigue resistance. Yet, issues may also appear with strain-control tests, with one possibly observed in this study being that stiffer samples may perform better.

To avoid creep and viscoelastic flow, a possible strategy that may tackle this issue is performing the tests at a lower temperature (lower than 15 °C), low enough to make sure that the sample is more in its elastic region (δ < 45°). Testing the sample at a temperature condition that results in a behaviour closer to its viscous region (δ > 45°) may lead the sample to fail predominately due to flow [39,40]. Lowering the binder content can also overcome this issue, making the mortar sample stiffer, but care should be taken that the samples are workable enough. Nevertheless, a balanced temperature selection should be respected, where the temperature should be low enough to ensure elastic behaviour but also high enough so that it belongs in the intermediate temperature regime, where fatigue cracking is expected to occur.

Comparing the two scales, mortar and asphalt, there is no consensus concerning which RAB replacement rate will perform better in fatigue. The difference in ranking can be first explained by the blending of the RAB. In the mortar scale, the RAB blended fully with the virgin counterpart, whereas in bulk scale, the results pointed out that especially for the 70% RAB, there was lower RAB activity and thus lower blending efficiency. A second parameter that may affect the results, and consequently the ranking, is the mechanism that drove the mortar samples to failure at higher stress levels, which may be attributed to creep deformation, a failure mechanism not expected for the much stiffer asphalt samples. Evaluating the fatigue resistance of mixtures containing RA, the bulk scale (asphalt) is still the best approach to assess fatigue. Two main reasons are, first, that partial blending is most likely expected to occur and, second, the fact that fatigue can be initiated due to both cohesive and adhesive failure. The current fatigue mortar testing does not consider these aspects.

## 6. Conclusions and Recommendations

In this study, the effect of a RAB on the fatigue resistance of bituminous mortars and mixtures was investigated. Three mortar and asphalt mixtures, containing three RAB replacement rates 0%, 40%, and 70%, were assessed. Based on this investigation, the following conclusions were drawn concerning the two main objectives: the effect of a RAB on the fatigue resistance of asphalt mixtures and mortars and the effectiveness of only treating them with a virgin binder.

Concerning the impact of a RAB on the fatigue resistance:

- For the asphalt scale, following the ε_6_ rule, the 40% RAB mix performed better compared to the other mixtures. Based on the PV approach, the same asphalt mix (40% RAB) showed the highest fatigue resistance for the 125 μm/m level. In contrast, it changed at the 85 μm/m level, where the 0% RAB mix showed the highest fatigue resistance.
- For the mortar scale, the samples with more RAB yielded lower fatigue life, as exhibited by both conventional and energy approaches.

Concerning the effectiveness of the selected RAB treatment strategy:

- Based on the results of the asphalt scale, adding only the virgin binder was an effective approach for the 40% RAB mix, but not for the 70% RAB mix, as demonstrated by the weaker fatigue resistance.
- On the other hand, based on the mortar scale results, adding only the virgin binder was not beneficial for the mortars containing a RAB. The fatigue results seemed to be strongly influenced by the |G*| of the mortar samples, which was lower for the 40% and 70% RAB mortar mix.

Generally, evaluating the fatigue resistance of mixtures containing RA, the bulk scale (asphalt) is still the best approach to assess fatigue. The motivation is mainly that partial blending cannot be captured by the mortar tests, at least not when using the proposed testing method. Additionally, fatigue can occur due to both cohesive and adhesive failure, and the current fatigue mortar testing considers only the former. However, mortar fatigue tests are useful alternative tests for preliminary evaluation instead of performing binder fatigue tests.

The results of this study should be further validated for different RA types and with different treatment strategies (e.g., use of rejuvenators). Concerning the considered analysis methods, the RDEC approach is a promising alternative to assess fatigue resistance, as it has the advantage of being independent of material and testing conditions. However, the disadvantage of this method is that it requires an extended postprocessing effort. For the fatigue characterisation of mortars, certain adjustments were performed on the bitumen content to achieve a workable bituminous mortar mixture. This may influence the fatigue results; therefore, this factor needs further validation. A leaner mortar mixture may be a better representation of the actual mortar scale; however, that will require compaction aid to achieve a good mortar mixture. 

## Figures and Tables

**Table 1:** Fractional composition of the three bituminous mortar and APO-B mixtures for the three replacement rates: 0% RAB, 40% RAB, and 70% RAB.

–	Bituminous Mortar (BM) (<0.5 mm)			Asphalt Mixture (APO-B)		
Component	BM 0% RAB (%)	BM 40% RAB (%)	BM 70% RAB (%)	APO-B 0% RAB (%)	APO-B 40% RAB (%)	APO-B 70% RAB (%)
Limestone 6.3/14	–	–	–	26.29	15.84	7.97
Limestone 2/6.3	–	–	–	27.62	23.45	20.49
Limestone 0/2	25.83	20.56	15.06	26.25	20.92	15.84
Riversand 0/1	20.16	7.11	1.20	6.56	2.32	0.40
RA 0/14	-	20.73	34.61	–	29.48 ^*1^	50.88 ^*1^
RA Filler	-	8.88	14.83	–	–	–
Filler (type V28/38)	28.41	17.10	9.56	9.16	5.51	3.18
Bitumen	25.61	25.59	24.74	4.12	2.47 ^*2^	1.24 ^*2^
Total	100.00	100.0	100.00	100.00	100.00	100.00
^*1^: This part considers both the RA aggregates and binder ^*2^: Only the added virgin binder						

**Table 2:** Volumetric properties of the asphalt mixes.

Mixture	Maximum Density (g/m^3^) EN 12697-5	Air Voids (%) EN 12697-8
APO-B 0% RAB	2499.1	4.2 (± 0.9)
APO-B 40% RAB	2488.6	4.0 (± 0.7)
APO-B 70% RAB	2482.3	5.0 (± 0.8)

**Table 3:** Theoretical binder mix design and measured conventional properties of the final binder blends.

Code Name		0% RAB	40% RAB	70% RAB
Composition	RA binder (%)	0	40	70
	Virgin binder (%)	100	60	30
Penetration	RA (0.1 mm)	24.0	24.0	24.0
	Virgin binder (0.1 mm)—control	42.0	-	-
	Virgin binder * (0.1 mm)—HB	-	61.0	-
	Virgin binder * (0.1 mm)—SB	-	-	155.0
	Final binder blend (0.1 mm)	42.0	42.0	42.0
Tested binder properties of the final blends	Penetration (0.1 mm)	42.0	40.0	37.0
	Softening point (°C)	53.4	54.6	56.4

* For the 40% and 70% RAB mixes, the virgin binder added is an artificial blend of two other binders, the hard blend (HB) and soft blend (SB), respectively. The compositions of the artificial virgin binder blends are presented in Table 4.

**Table 4:** Virgin binder blends.

Code Name		Control	HB	SB
Virgin Binder Blend Composition	Component 1 (binder type)	35/50 (100%)	50/70 (72.4%)	50/70 (16.4%)
	Component 2 (binder type)	-	70/100 (27.6%)	160/200 (83.6%)
Penetration	Component 1 (0.1 mm)	42.0	55.0	55.0
	Component 2 (0.1 mm)	-	80.0	190.0
	Virgin binder blend (0. 1mm)	42.0	61.0	155.0

**Table 5:** Fatigue test parameters for the asphalt mixtures.

$N_{f,50}=A{\left(\varepsilon \right)}^{B}$	A	B	R^2^ (%)	ε_6_ (μm/m)	ε_5_ (μm/m)
APO-B 0% RAB	2 × 10^8^	−6.121	95.62	102	145
APO-B 40% RAB	2 × 10^15^	−4.452	93.66	104	170
APO-B 70% RAB	2 × 10^16^	−5.282	91.78	94	140

**Table 6:** Fatigue test parameters of tested mortar mixtures.

$N_{f,GxC}=A{\left(\tau \right)}^{B}$	A	B	R^2^ (%)
APO-B 0% RAB	17377	−7.143	94.15
APO-B 40% RAB	11545	−6.498	95.46
APO-B 70% RAB	10078	−6.912	88.06
